# Hitchhiker’s Guide to *Borrelia burgdorferi*

**DOI:** 10.1128/jb.00116-24

**Published:** 2024-08-14

**Authors:** Jeffrey S. Bourgeois, Linden T. Hu

**Affiliations:** 1 Department of Molecular Biology and Microbiology, Tufts University Lyme Disease Initiative, Tufts University School of Medicine, Boston, Massachusetts, USA; University of Massachusetts Chan Medical School, Worcester, Massachusetts, USA

**Keywords:** Lyme disease, *Borrelia*, *Borrelia burgdorferi*, *Borreliella burgdorferi*, genetics, immunology, host-pathogen interactions, history, spirochetes

## Abstract

Don’t Panic. In the nearly 50 years since the discovery of Lyme disease, *Borrelia burgdorferi* has emerged as an unlikely workhorse of microbiology. Interest in studying host-pathogen interactions fueled significant progress in making the fastidious microbe approachable in laboratory settings, including the development of culture methods, animal models, and genetic tools. By developing these systems, insight has been gained into how the microbe is able to survive its enzootic cycle and cause human disease. Here, we discuss the discovery of *B. burgdorferi* and its development as a model organism before diving into the critical lessons we have learned about *B. burgdorferi* biology at pivotal stages of its lifecycle: gene expression changes during the tick blood meal, colonization of a new vertebrate host, and developing a long-lasting infection in that vertebrate until a new tick feeds. Our goal is to highlight the advancements that have facilitated *B. burgdorferi* research and identify gaps in our current understanding of the microbe.

## INTRODUCING *BORRELIA BURGDORFERI*, AN ATYPICAL MODEL SYSTEM

We begin this review with a simple truth: *B. burgdorferi* is unusual. Unlike many of its prokaryotic cousins featured in the flagship series (1–6), each spirochete houses several copies of both its linear chromosome and numerous other replicons (both circular and linear) (7–9)—many of which can be shed sporadically during laboratory cultivation (10). Analysis of the *B. burgdorferi* genome shows evidence of genome shrinkage, including loss of core metabolism pathways (11), which aligns both with its obligate host-associated lifestyle and with the highly enriched media required to keep the microbe growing in laboratory settings. Furthermore, no traditional virulence factors have been discovered, suggesting that *B. burgdorferi* has a very limited repertoire to manipulate or evade host immunity during infection. Yet, despite these limitations, *B. burgdorferi* is able to stably colonize rodent (12–14) and tick (15) hosts for months to years.

These unique attributes notwithstanding, *B. burgdorferi* has become a “model organism” for understanding other bacteria that occupy similar niches. *B. burgdorferi* is well-studied among spirochetes (searching PubMed on 8 May 2024 revealed 11,465 results for *B. burgdorferi* or *Borreliella burgdorferi*, 3,489 results for *Leptospira interrogans*, and 6,886 results for *Treponema pallidum*), and some researchers have used the microbe as a launching point to understand cell biology in the *Spirochaetes* phylum, particularly the structure and function of periplasmic flagella [reviewed in references (16–18)]. These unique flagella are important to many key aspects of spirochete biology, and in *B. burgdorferi*, they enable its characteristic spiral shape (19) and colonization of vertebrate and invertebrate hosts (20, 21). *B. burgdorferi* has also served as an effective ectopic expression system to understand *T. pallidum* protein biology and immunogenicity (22, 23). Beyond spirochetes, *B. burgdorferi* is also a general model of vector-borne disease—the relative ease of studying *B. burgdorferi* discussed below makes it an attractive option for understanding pressures on microbes during invertebrate-vertebrate-invertebrate transmission. However, as with all models, some caution should be used when overinterpreting “universal” lessons from *B. burgdorferi*. While Monod and others have noted that “What’s true for *Escherichia coli* is true for the elephant” to describe the robustness of the *E. coli* molecular biology model (3), when trying to compare biology across bacterial species, one may be better served considering George E. P. Box’s famous advice: “All models are wrong, but some are useful.”

In the text below, we will discuss the identification of *B. burgdorferi*, key developments in the field, and what we have learned about *B. burgdorferi* enzootic cycling and pathogenesis over the last 50 years.

## DISCOVERY OF *B. BURGDORFERI* AND ITS ENZOOTIC CYCLE

### Identification of a tick-borne, spirochetal illness

**Fig 1:**
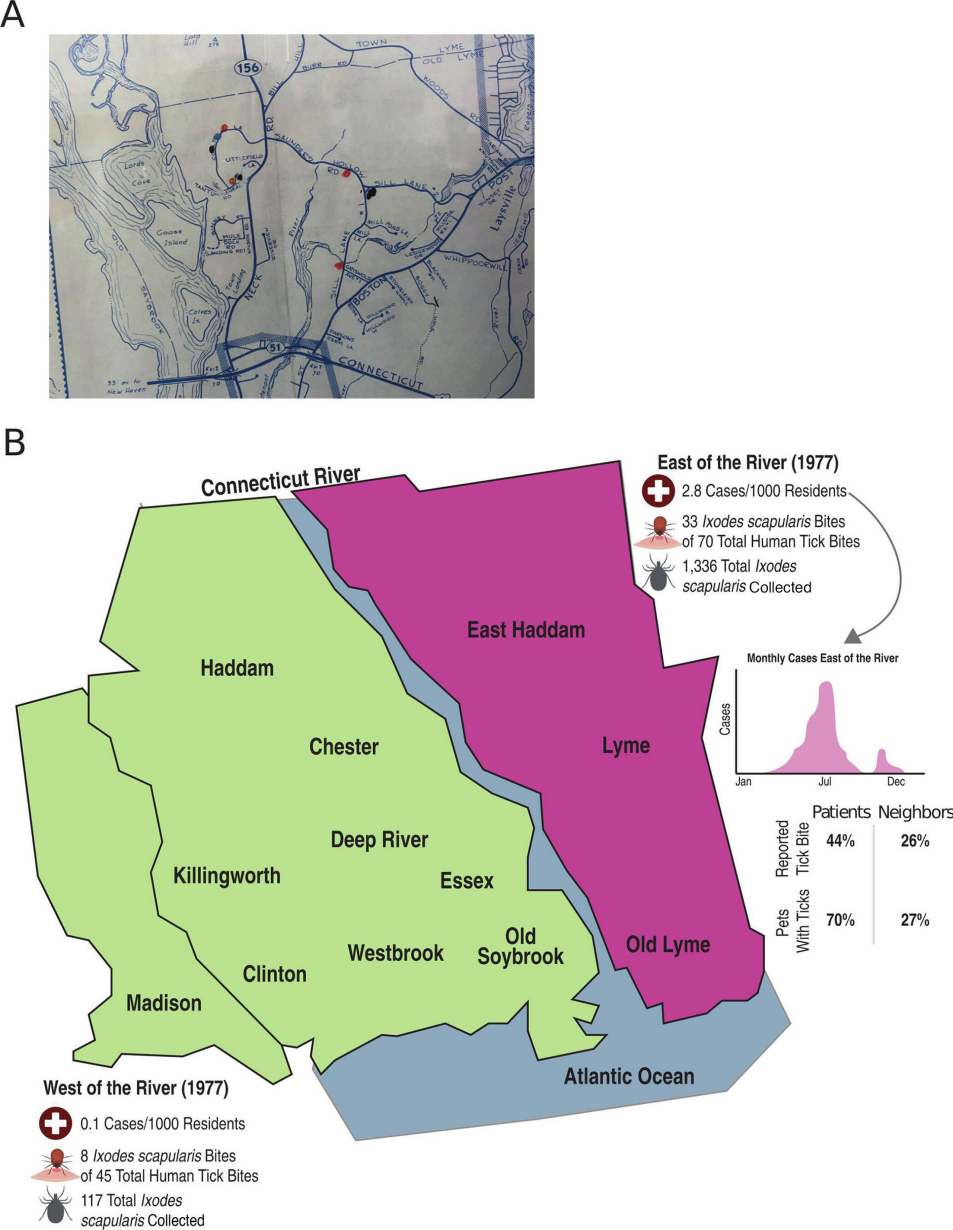
Epidemiological studies identify a cluster of *Ixodes* scapularis-associated arthritis in the Lyme, Connecticut, region. (**A**) Original mapping of cases in Old Lyme, Connecticut, performed by Dr. David Snydman after being deployed as an Epidemic Intelligence Officer. Reproduced with permission from Dr. Snydman. (**B**) Work by Steere and colleagues demonstrated a clear regional bias for cases east of the Connecticut River, a temporal bias for summer and fall months, and patients with Lyme disease had increased interactions with ticks (26). Work by Wallis and colleagues demonstrated that *I. scapularis*, specifically, was far more common in humans and other mammals (deer, *Peromyscus* rodents) east of the river (27).

The identification of Lyme disease began with a cluster of arthritis among otherwise healthy children in Old Lyme and Lyme, Connecticut in the early 1970s (24). Early reports from Polly Murray and Judith Mensch, who had noted unusual symptoms in children and local families, led to the Centers for Disease Control and Prevention sending a young Epidemic Intelligence Officer, Dr. David Snydman, to investigate. Given the rheumatological symptoms noted, Dr. Snydman requested assistance from Yale University and involved a rheumatology fellow, Dr. Allen Steere, on the team. Following the mapping of reported cases (Fig. 1A), painstaking epidemiological work determined that juvenile rheumatoid arthritis, a genetic disorder with an attack rate of 1 in 10,000 (25), was unlikely to be the cause of the cluster, and they quickly focused on potential infectious causes. The pattern of disease was most consistent with a vector-borne disease (24). A key clue in the investigation that would eventually lead to the discovery of the causative organism was that approximately one-quarter of the afflicted children had recalled a distinctive circular or oval rash prior to the onset of arthritis.

During a presentation of some of these patients at a dermatology case conference at Yale, there was a serendipitous event where a visiting Danish dermatology resident noted that the rash was similar to those seen in Europe in patients with a tick-borne disease called Bannwarth’s syndrome (personal communication, Allen Steere). The European patients often presented with a rash called erythema migrans (EMs). The association between European EM and *Ixodes* ticks had been established by Afzelius in 1909 (28), and the connection between a rash (presumed to be erythema migrans), neurological illness, and *Ixodes* was made by 1922 (29). One 1951 report from Sweeden reported “using the spirochetal stain evolved by him, Lennhoff has succeeded in demonstrating organisms resembling spirochaetes in biopsy specimens taken from the erythematous lesions” (30). This led to penicillin, which was effective for the treatment of another spirochetal disease, syphilis, becoming a common and effective treatment for EM (30, 31). While the European patients did not have the arthritis seen in the cases in Old Lyme, the similarity to EM led to additional epidemiological work by Steere, Robert Wallis, and others that implicated the “deer tick” or “black-legged tick” *I. scapularis* (formerly also called *Ixodes dammini*), as the probable vector of “Lyme disease” (Fig. 1B) (26, 27).

Following the identification of a probable vector of disease, Steere and others began attempting to treat Lyme disease with penicillin and found that it reduced both the duration of EM and the likelihood of developing arthritis (32). During a presentation of the data for the treatment of Lyme disease with penicillin, Dr. Alan Barbour, a young infectious disease physician who was about to start a post-doctoral fellowship at the NIH’s Rocky Mountain Laboratories studying spirochetes, became interested in the disease. He convinced his mentor, Dr. Willy Burgdorfer, to look for spirochetes in ticks from Lyme disease endemic areas that had been collected and sent by Dr. Jorge Benach at Stony Brook University. This led to the identification of a spirochete found in ticks from endemic regions in 1982 (33) that would later also be isolated from Lyme disease patients (34). For a more comprehensive description of the history surrounding the identification of *B. burgdorferi*, we refer the reader to a recent first-person narrative by Drs. Barbour and Benach (35).

Following the discovery of *B. burgdorferi* in North America, EMs (and arthritis, carditis, and neurological illnesses that follow them) in both North America and Europe were confirmed to be caused by *B. burgdorferi* spirochetes (36, 37). Notably, while most North American Lyme disease is caused by *B. burgdorferi sensu stricto* [recently changed to *Borreliella burgdorferi* based on genomic analyses (38), though some researchers have objected to the new name (39)], European Lyme disease is caused by a collection of related genospecies called *B. burgdorferi sensu lato* (which includes *B. burgdorferi sensu stricto*) (40). All further use of the term *B. burgdorferi* in this review will refer exclusively to *B. burgdorferi sensu stricto*.

Focusing on North American Lyme disease, it is interesting to contrast the gaps in time between the recruitment of health officials to Lyme, Connecticut (1975), the first peer-reviewed manuscript on Lyme disease (1977) (24), the implication of *I. scapularis* as the vector (1978) (26, 27), and the tentative identification of a causal agent (1982) (33) with the 12-day separation between the first reports in 2019 of pneumonia in Wuhan, China and the release of the SARS-CoV-2 genome. Technological advances in metagenomic sequencing account for a large component of the different timescales here—however, additional factors likely slowed early research on *B. burgdorferi*. The organism is sparse in most sites in infected humans—particularly in the joint where live organisms have never been recovered (a single prior report proved to be erroneous)—and requires very specific growth culture media to grow *in vitro*, making it difficult to fully fulfill Koch’s postulates even with modern technologies.

### Understanding *B. burgdorferi* spread in nature

After confirming *I. scapularis* [and its west coast North American cousin *I. pacificus* (41)] were the primary culprits in spreading Lyme disease in North America, a key question became how the ticks themselves become colonized with *B. burgdorferi. B. burgdorferi* transovarian (mother-to-offspring) spread does not occur to any significant degree (42), though early studies did observe some passage of spirochetes across generations that led to confounding narratives (15, 43). It was subsequently recognized that *Ixodes* ticks can be co-colonized with the transovarially passed spirochete, *Borrelia miyamotoi* [summarized in reference (42)]. Notably, even though *B. burgdorferi* was originally believed to have some tick-to-tick spread, Burgdorfer and others noted in their work that the rates of viable spirochetes passed to offspring were far too low to account for the abundance of *B. burgdorferi* in nature. This paired with other research from the same period that noted extremely high (“universal,” according to the original authors) *B. burgdorferi* colonization of the white-footed mouse *Peromyscus leucopus* (44), which is highly abundant in North America and serves as one of the critical hosts for *Ixodes* ticks, particularly larvae in the northeastern and midwestern United States (45), led to a hypothesis that ticks acquire *B. burgdorferi* from vertebrate reservoirs. Later work would experimentally confirm *P. leucopus* “reservoir competency”—or the ability for *P. leucopus* to become colonized by and spread the spirochete to ticks (13). While numerous models and studies demonstrate that *P. leucopus* is a critical reservoir species for *B. burgdorferi*, it is not the only important reservoir (46–49). Notably, shrews may help to promote *B. burgdorferi* abundance (46, 47)—particularly in the absence of high *P. leucopus* density. Other species such as squirrels can “dilute” *B. burgdorferi* abundance by feeding ticks without enabling *B. burgdorferi* spread (46). Furthermore, certain strains of *B. burgdorferi sensu stricto* (50) and *sensu lato* (51), particularly *Borrelia garinii* which is found in Europe, appear better adapted to birds than to *Peromyscus*.

**Fig 2:**
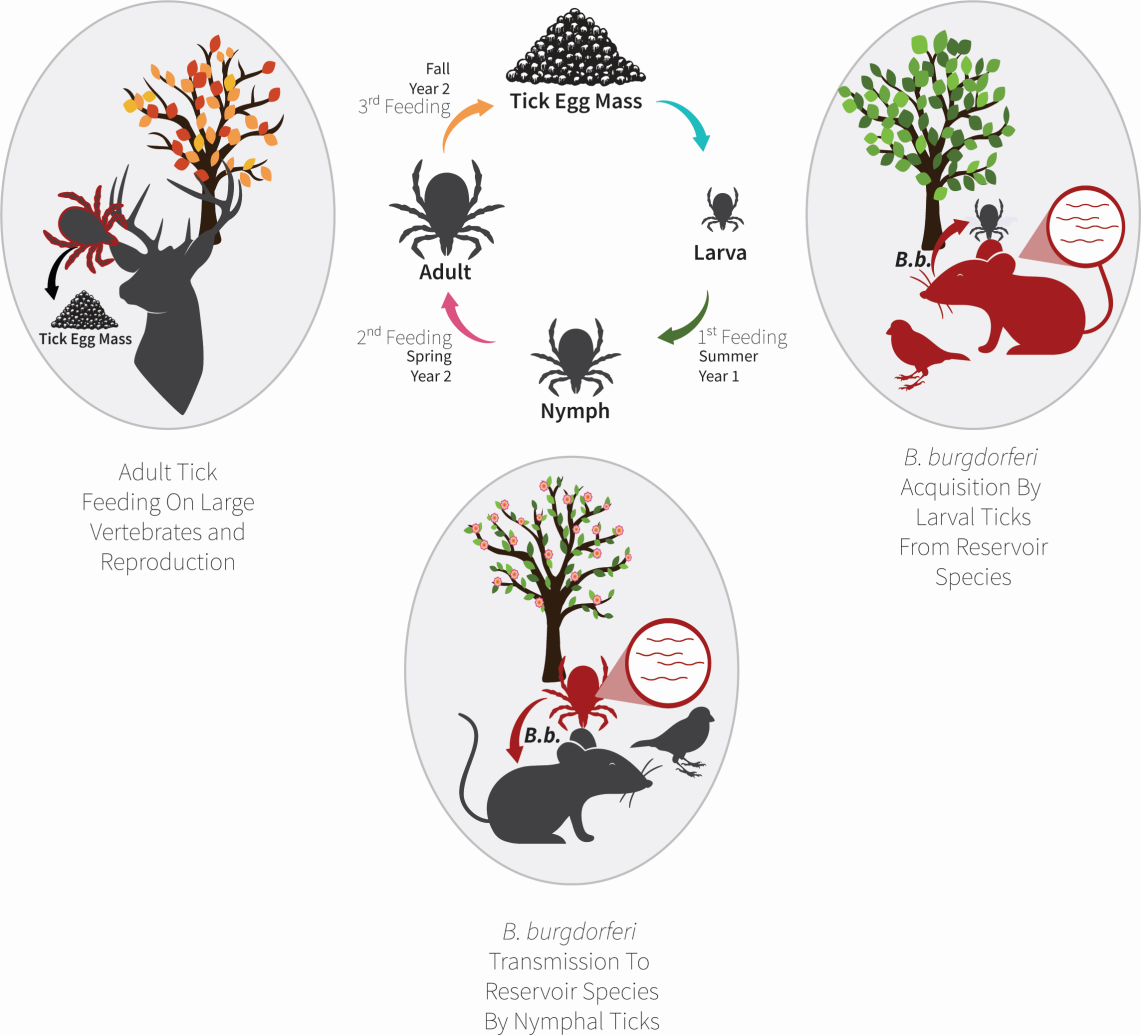
Understanding *B. burgdorferi* spread in nature. *B. burgdorferi* vectors, *Ixodes* ticks, take three blood meals throughout their lifetimes over the course of 2 years: once as larvae, once as nymphs, and once as adults. In spring, the nymphal *I. scapularis* blood meal enables *B. burgdorferi* to spread into reservoir hosts, typically small mammals or birds. Later in the year, larvae feed on these colonized reservoirs, which results in the acquisition of the spirochete by new ticks. Finally, in the fall adult *Ixodes* feed on larger vertebrates. Notably, the infection status of the tick has very little impact on the spread of *B. burgdorferi* in the adult stage, as the hosts that adult *I. scapularis* feed on are typically non-permissive or dead-end hosts for *B. burgdorferi*. Instead, this stage is important for the continued propagation of the tick vector. Red animals represent *B. burgdorferi* colonization, and the red arrows represent the direction of *B. burgdorferi* spread at each tick life stage. The adult tick is represented as both gray and red to represent that both colonized and *B. burgdorferi*-free adult ticks contribute to *I. scapularis* reproduction.

There is no reproducible evidence supporting mammal-to-mammal *B. burgdorferi* spread. This means that in order for *B. burgdorferi* to persist, it depends on an enzootic cycle, through which *B. burgdorferi* continuously cycles between its vertebrate reservoir species and invertebrate vectors (Fig. 2). Thus, *B. burgdorferi* must routinely adapt to and persist in dramatically different hosts—while only utilizing fewer than 1,400 protein-coding genes.

## KEY MILESTONES IN MODELS OF LYME DISEASE

**Fig 3:**
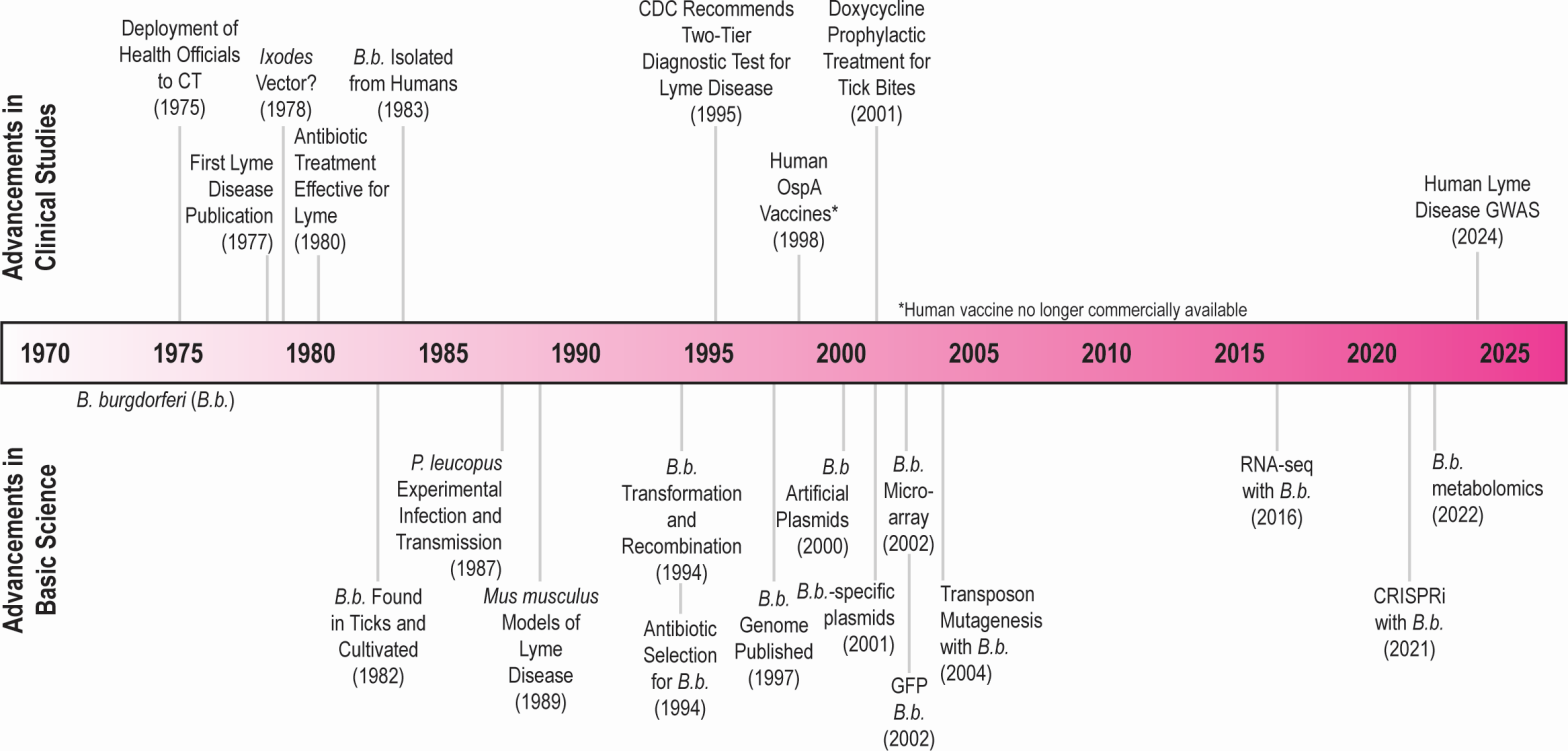
Advances in *B. burgdorferi sensu stricto* (*B.b*.) research. Clinical advances in *B. burgdorferi* research include the first research publication on Lyme disease (24), the suggestion that it was transmitted through *Ixodes* ticks (26, 27), the first use of antibiotics to treat the disease (32), isolation of *B. burgdorferi* from humans (34), standardization of Lyme disease testing (52), the development of human OspA vaccines (53, 54), the use of doxycycline as a prophylactic treatment (55), and completion of a Lyme disease human genome-wide association study (56, 57). Basic science advances in *B. burgdorferi* research include identification of the spirochete in ticks (33), experimentally modeling the *B. burgdorferi* enzootic cycle with *P. leucopus* (13), development of the first *Mus musculus* models for Lyme disease (58, 59), transformation of DNA into *B. burgdorferi* for recombination (60), cloning of genetic mutants by antibiotic selection (60), publication of the first genome drafts (7, 8), transformation of the first autonomously replicating artificial plasmid (61), generation of stable *B. burgdorferi-*specific shuttle vectors (62, 63), effective use of GFP in *B. burgdorferi* (63, 64), development of a *B. burgdorferi* DNA microarray (65, 66), use of genome-wide transposon mutagenesis for *B. burgdorferi* (67), the use of RNA-sequencing (RNA-seq) for *B. burgdorferi* (68–70), CRISPRi in *B. burgdorferi* (71), and the use of metabolomic technologies with *B. burgdorferi* (72).

Since the discovery of Lyme disease in Connecticut, substantial progress has been made in understanding *B. burgdorferi* pathogenesis and bacteriology (Fig. 3). In the text below, we will discuss some of the most notable advances that have enabled *B. burgdorferi* study.

### Cultivation of *B. burgdorferi in vitro*

A major development in the study of *B. burgdorferi* was the ability to cultivate the spirochete from ticks in 1982 using a modified Kelly’s medium, which included the addition of CMRL-medium (developed at **C**onnaught **M**edical **R**esearch **L**aboratories) and yeastolate (33). In his historical perspective (35), Alan Barbour notes that the formulation of this modified (originally “fortified”) Kelly’s medium (73) was critical for growing the bacteria from ticks. This highlights the lengths early researchers went to in order to culture *Borrelia* spp., including providing substantial nutrient supplementation (including serum, bovine albumin, N-acetylglucosamine, and glucose), adding gelatin which modifies *B. burgdorferi* motility behavior, and minimization of oxygen exposure and carbon dioxide loss [reviewed in reference (74)]. These formulations have been continuously improved, leading to two predominant media formulations: Barbour-Stonner-Kelley Medium II (BSK-II) (75) and a commercially available derivative BSK-H (76). Notably, the complexities of these media have led to a variety of problems. First, batch-to-batch variability plagues both BSK-II and the commercial BSK-H [for example, see references (77, 78)], meaning media must undergo quality control prior to use (79). Second, for reasons that remain obscure, differences in BSK-II and BSK-H drive numerous differences in *B. burgdorferi* biology at the molecular level (80) and in the ability to infect rodents (78). Despite the technical ease of BSK-H, many groups—including the authors of this review—remain committed to making their own BSK-II based on higher rates of successful *B. burgdorferi* cultivation.

The ability to culture an otherwise obligate host-associated bacterium *in vitro* unlocks substantial experimental manipulation, ranging from direct observation and manipulation of the microbe for microbiological assays to being able to prepare clonal isolates of bacteria for animal challenge. However, BSK-based media are complex, and the exact requirements for *B. burgdorferi* cultivation remain unknown. This makes it difficult to modify the medium to examine specific questions involving metabolism, though some success has been had removing specific components (e.g., glucose) and adding alternative components (e.g., chitobiose) in excess (81). Other studies have attempted to biochemically alter certain components of the medium [e.g., delipidation of bovine serum albumin, serum, and yeast extract (82) and chelation of metals (83)] in order to ask targeted metabolic questions. While functional, a deeper understanding of *B. burgdorferi* biology may require the development of a truly defined medium more amenable to biochemical and metabolic experiments—though this has not been successful despite attempts by multiple groups.

### Establishing animal models infection with *B. burgdorferi*

Soon after the successful culture of *B. burgdorferi in vitro*, attempts to test and develop animal models of infection were initiated. *B. burgdorferi* was found to be capable of infecting multiple small animals including but not limited to laboratory strains of inbred *Mus musculus* (58, 59), rats (84), *P. leucopus* (13), hamsters (13, 85, 86), gerbils (87, 88), and rabbits (33, 89, 90). The focus during these early studies was in finding an animal model that mimicked the stages of human Lyme disease; however, it was soon apparent that none of the animals could produce a perfect match. Among small mammals, only rabbits developed the classic erythema migrans rash seen in humans with early disease (33). Although all laboratory mice were able to be infected with *B. burgdorferi*, different strains of inbred mice developed varying levels of carditis and arthritis (58), and none developed significant signs of meningitis [during the early evaluations; subsequent studies have shown invasion of the dura mater of mice by the organism but minimal inflammatory signs (91)]. Three strains of mice became the dominant animal models for studying Lyme disease—C57BL/6, C3H, and BALB/c—each of which has particular characteristics that make it attractive as a model. C57BL/6 has been labeled as “resistant” to Lyme disease manifestations as they develop only mild carditis and arthritis in response to infection (58). C3H mice develop more severe arthritis and carditis (58), and BALB/c develops inoculum dose-dependent inflammatory responses to *B. burgdorferi* (92). Forward genetic studies have identified genetic loci involved in the differential inflammatory responses seen in C57BL/6 and C3H strains of mice (93, 94), which drive an increased type I interferon response in C3H mice (95), although the genes identified are not clearly the same as ones that may be involved in controlling the level of inflammation in humans. Of note, all mouse strains will largely resolve signs of inflammation without antibiotic treatment despite the continued presence of the organism (96). In some ways, these models parallel the course of Lyme disease in humans infected with *B. burgdorferi*. Before the adoption of antibiotics for treatment, it was noted that patients can spontaneously clear erythema migrans (32), as well as inflammatory manifestations of Lyme disease such as facial palsy and heart block (carditis) without antibiotics (97, 98). Some patients, particularly children, can also spontaneously resolve arthritis (24)—though this resolution can take up to 5 years and recurrences are common in the first years after the development of Lyme arthritis.

Rhesus macaque (*Macaca mulatta*) has been utilized as a model for Lyme disease, as early studies noted that the monkeys develop both early and late signs of infection that are similar to human disease with some infected animals displaying erythema migrans, transient bradycardia, and infiltration of immune cells into cerebral spinal fluid (99). Unlike rodent models, some *M. mulatta* appear to develop signs of neuroborreliosis, including lethargy, peripheral neuritis, and peripheral demyelination (99, 100). The variability in these symptoms may recapitulate the variability seen in human manifestations of the disease. There have also been attempts to use *M. mulatta* for the study of antibiotic-refractory disorders (101, 102); however, it remains unclear whether non-human primates are suitable models for these disease manifestations (103).

Additional work has utilized laboratory models to study the interactions of *B. burgdorferi* with its native hosts where colonization generates minimal to no symptoms. *P. leucopus* and *Peromyscus maniculatus* have near identical husbandry requirements to *M. musculus*, making them an approachable and realistic model for enzootic cycling (13, 104–110) as well as host-tick interaction studies (111, 112). These studies have been aided by a growing number of repositories and stock centers that can provide *Peromyscus* and *Ixodes* ticks to labs for enzootic studies. The study of avian (106, 113–116) and reptilian (117, 118) hosts and their reservoir capacity for *B. burgdorferi* and *B. burgdorferi sensu lato* strains are less common due to their more unique husbandry requirements but are growing in usage.

Regardless of species, laboratory infection of animals with *B. burgdorferi* typically occurs through one of two methods: needle injection of a pure culture (typically via subcutaneous or intradermal injection to mimic a tick bite, though other injection schema including intraperitoneal and intraarticular injections do also result in successful infection) or use of *B. burgdorferi-*colonized nymphal *I. scapularis*—which are typically generated by feeding uninfected larvae on needle infected rodents and housing the ticks through their first molt. Alternatively, ticks can be artificially colonized with *B. burgdorferi* through immersion in a pure culture and then subsequently used to infect animals (119). Needle infection, while more artificial, is substantially more approachable and less time consuming than tick-based infections and often does successfully uncover phenotypes associated with bacteria transmitted by the tick [for example, efficacy of OspA-based vaccines against *B. burgdorferi* infection (120) or the requirement for *ospC* during infection (121)]. However, this is not universally the case, and numerous genes have been identified to be required for *B. burgdorferi* virulence when transmitted by a tick but not when transmitted by needle [for instance *bba07* (122)]. Differences in bacterial gene expression in *in vitro* grown vs tick grown *B. burgdorferi* and differences in inoculating doses may explain some of these disparities (123, 124), which are supported by very high infectivity of “primed” *B. burgdorferi* isolated from fed ticks (125). Additionally, tick-host interactions also change the requirements for *B. burgdorferi* survival during infection. During tick-mediated infection, *Ixodes* salivary proteins that have immunomodulatory properties [discussed further below and recently reviewed in reference (126)] are injected into the host alongside the bacteria—meaning that *B. burgdorferi* at a needle injection site are experiencing a dramatically different immune landscape than those transmitted by ticks. An interesting case where needle inoculation led to erroneous conclusions was the development of decorin-binding protein A (DbpA) as a vaccine candidate. While vaccination with DbpA protected against needle infections with *B. burgdorferi*, it failed to protect against infection through feeding of colonized ticks due to the lack of expression of DbpA while the organism resides in the tick—leading to the failure of a vaccine that had progressed to advanced stages of development (127). As with all models, choosing the correct animal host and infection model requires assessing the costs and benefits of each option, informed by the specific question being asked in each individual experiment.

### Deciphering the *B. burgdorferi* genome

The first *B. burgdorferi* genome was partially sequenced in 1997 (7), though it would take until 2000 before the final extrachromosomal elements were fully sequenced (8). This initial genome provided the framework to study the roughly 900 kilobase linear chromosome and over 600 kilobases of linear and circular plasmids [spread across 21 plasmids in the originally sequenced strain B31 (8)]. The exact plasmid number and the exact genes located on each plasmid differs across *B. burgdorferi* strains [most recently demonstrated across 299 isolates in (128)] and even different *in vitro* passaged lineages can differ in plasmid content due to spontaneous plasmid loss (10). Another notable aspect of the genome is that it is highly adenine and thymine rich, with the chromosome containing 28.6% guanine and cytosine content and the plasmids ranging from 23.1% to 32.3% guanine and cytosine content. This can lead to challenges when expressing non-native genes in *B. burgdorferi* as careful codon optimization is necessary to avoid toxicity (129) and enable expression (130).

Current bioinformatic tools estimate that there are 1,391 protein-coding genes, 37 RNA genes, and 135 pseudogenes present across the B31 chromosome and plasmids (131), as well as over a thousand small non-coding RNA (68, 69). Of these, 124 genes (8.9%) are predicted to be lipoproteins (132, 133), which is exceptional when compared to the predicted ~90 lipoproteins present among the ~4,328 protein-coding genes in the *E. coli* genome (2.1%) (134). Of the *B. burgdorferi* lipoproteins, 88% (79 proteins) of studied plasmid-encoded lipoproteins appear to traffic to the *B. burgdorferi* surface, while 19% (7 proteins) of studied chromosomal lipoproteins are present on the surface, and only 39 lipoproteins in total were found to traffic to the periplasm (132). It should be noted that this study did depend on the overexpression of each lipoprotein, which could affect localization. However, if this is an accurate representation of non-overexpressed lipoproteins, this differs from *E. coli* where most lipoproteins are found in the periplasm (134). This pairs with experimental work that has highlighted a key role for lipoproteins in interfacing with vertebrates and invertebrates during the enzootic cycle [reviewed in reference (135)]. In striking contrast to the high abundance of lipoproteins, *B. burgdorferi* lacks many metabolic pathways (7, 11), including pathways for the tricarboxylic acid cycle, oxidative phosphorylation, fatty acid synthesis and degradation, amino acid synthesis, the urea cycle, polyamine synthesis, and nucleotide synthesis. Instead, *B. burgdorferi* encodes an array of transporter proteins that appear to capture critical nutrients from its environment. This aligns well with the abundant nutrients required for the cultivation of the microbe *in vitro*.

While sequencing the B31 genome provided a wealth of information, studies predating the assembled genome had spent considerable effort quantifying the number of plasmids present in *B. burgdorferi* (136)—including the identification of several highly related circular plasmids that are each roughly 32 kilobases [circular plasmid (cp)32] (137, 138). This finding was surprising given the overall minimal *B. burgdorferi* genome. Later studies would go on to confirm hypotheses proposed by Casjens et al. (138) that this plasmid family encodes a prophage (139) which is produced in the tick host (124) and coupled to RpoS expression (140). Recently, it was confirmed that these cp32 phages could not only transduce cp32 (141) but also other plasmids and even regions of the linear chromosome (142)—indicating a potential mechanism for horizontal gene transfer in *B. burgdorferi*.

### Manipulating the *B. burgdorferi* genome

*B. burgdorferi* is, with some effort, a genetically tractable organism. The history and nuances behind genetic manipulation in *B. burgdorferi* have been thoroughly reviewed elsewhere (143), but it is worth noting that many of the advances are rooted in discoveries made by investigators at the Rocky Mountain Laboratories at the National Institutes of Allergy and Infectious Diseases [select examples include references (60, 62, 67, 144–146)] who were given the freedom to take on these difficult and time consuming tasks that ultimately allowed the field to make major leaps forward. Briefly, the first genetic manipulation of the spirochete was described in 1994 by Samuels and colleagues following the introduction of a coumermycin-resistance allele into the chromosome via homologous recombination (60). Spontaneous single-point mutations leading to resistance to coumermycin made it a difficult selectable marker to use, but it was subsequently followed by other more durable resistance markers in the aminoglycoside class. The first autonomously replicating artificial *B. burgdorferi* plasmid was described in 2000 (61) using a broad-host-range shuttle vector, though the vector has a variety of issues that have made it unpopular for use. Instead, seminal work in 2001 by Stewart et al. described the first *Borrelia*-specific shuttle vector by incorporating aspects of the endogenous cp9 plasmid into the artificial construct (62). Shortly thereafter, a second plasmid was generated using a region of cp32 (63). These vectors have stood the test of time and are cornerstones of *B. burgdorferi* molecular biology. These and other genetic manipulations can be introduced through electroporation using very high concentrations of DNA (147, 148), although plasmids are often lost during passage in ticks or mammals in the absence of additional strategies (121, 149). While it is possible to transform plasmids into fully wild-type *B. burgdorferi*, restriction-modification systems in *B. burgdorferi* act as a substantial barrier to transformation, particularly the predicted type IV restriction-modification enzymes carried on linear plasmid (lp)25 and lp56 (*bbe02* and *bbq67*, respectively) (150–153). Even in strains where one or both of these plasmids are initially present, the transformation of recombinant plasmids can inadvertently select for clones that have lost these barriers to transformation (150). While lp56 is dispensable for rodent infections (154), lp25 is required for both tick colonization and rodent infection due to the nicotinamidase gene *pncA* (*bbe22*) (154, 155). Other genes on lp25 are also required for tick colonization including [but likely not limited to (156)] *bptA* (*bbe16*) (157). Thus, many approaches for targeted mutagenesis use clones that are specifically disrupted for *bbe02*. Another strategy when genetically modifying the genome is to replace *bbe02* [for example, replacement of *bbe02* with *lacI* (158) or *luciferase* (159)]. Alternatively, groups exclusively interested in vertebrate stages of infection can transform *B. burgdorferi* lacking lp25 with plasmids containing *pncA* alongside genes of interest in order to enable (i) efficient transformation and (ii) long-term plasmid retention during murine infection due to the presence of *pncA* (149).

Transformation and homologous recombination have laid the foundations of molecular biology in *B. burgdorferi*: knock-out, knock-in, and complementation experiments are now feasible and, in fact, routine. The number of experiments possible with these techniques has grown with the number of tools optimized for *B. burgdorferi*, including inducible promoters [IPTG-inducible (130, 158), tetracycline-inducible (160), IPTG-use has been utilized *in vivo* (158, 161)], constitutive promoters (162), antibiotic resistance cassettes (144, 145, 162, 163), and fluorescent proteins (63, 64, 162, 164, 165). Additional advances in genetic manipulation include transposon mutagenesis (67) and more recently CRISPR-based technologies (71, 129, 166). The efficiency of transposon mutagenesis remains poor, and the largest transposon mutant library has transpositions in only approximately 790 genes, with many non-essential genes lacking insertions. However, even this suboptimal library has led to insights into gene function using transposon-sequencing (Tn-seq) strategies (167) to identify genes involved in diverse functions for the bacteria including carbohydrate utilization (168), reactive oxygen species or reactive nitrogen species resistance (169, 170), surviving the larval *I. scapularis* blood meal (171), and the role of sRNA in mouse infection (172). With the currently available tools, it is anticipated that a CRISPRi library may be available in the near future which will improve the ability to perform whole-genome loss-of-function screens.

### Modeling *B. burgdorferi* interactions with its hosts

Early attempts to understand how *B. burgdorferi* adapts to its different hosts were focused on changing the environment of *in vitro*-grown organisms and examining their responses. Alterations in growth conditions such as temperature and pH led to important insights into gene regulation by the organism (65, 66, 173–179) including one of the central tenets of *B. burgdorferi* biology—that different gene sets are expressed to survive in tick vs mammalian hosts. However, with improvements in technologies for studies of the organism *in situ*, it became clear that these simple changes to *in vitro* culture all failed to capture the complexity of the *B. burgdorferi* response to its native hosts (123).

BSK-media warmed to 37°C has served as a major model for “mammalian-like” gene expression, in part because *B. burgdorferi* is present at extremely low numbers in host tissue—making it difficult to directly measure gene expression in those tissues. Targeted assays (e.g., reverse transcription quantitative PCR [RT-qPCR]) have had some success measuring bacterial processes in rodent tissues (180), and these approaches have been expanded to a significant degree—including quantifying expression of 137 lipoprotein genes during mammalian infection (181). Another solution for transcriptomic screening in mammalian hosts utilized dialysis membrane chambers filled with media containing *B. burgdorferi* that were (i) embedded in the peritoneum of rats or mice, (ii) allowed to incubate in the rodent, and (iii) retrieved for gene expression measurement (65, 182, 183). This approach revealed numerous differences in gene expression compared to what was observed by culturing *B. burgdorferi* in BSK-media at 37°C (65, 183), underscoring that temperature is not the only signal *B. burgdorferi* senses in the vertebrate host. However, a drawback of the model is that it does not accurately mimic the effects of direct contact with cells. We note that, currently, no study has successfully leveraged untargeted RNA sequencing to measure global transcript abundance in mammalian tissues.

Transcriptomic studies in the tick lagged behind rodent studies. Again, the major obstacle was the low number of organisms found inside the tick, which made targeted approaches feasible (183–185) but untargeted approaches difficult. However, in 2015, Iyer et al. successfully employed amplification-based enrichment of *B. burgdorferi* transcripts with DNA microarray to examine gene expression in fed ticks (123). A more recent study combined antibody pulldown of whole bacteria followed by lysis and RNA sequencing to thoroughly map the *B. burgdorferi* transcriptome across different stages of tick feeding (124). A separate approach successfully enriched *B. burgdorferi* transcripts following RNA isolation using biotinylated probes to compare the transcriptome of wild-type *B. burgdorferi*, Δ*rpoS*, and strains with altered c-di-GMP synthesis or signaling in fed nymphs (161).

Another popular model of *B. burgdorferi* biology is measuring interactions with host cells *ex vivo* or *in vitro*. This falls into two categories: (i) stimulation of host cells with *B. burgdorferi* to understand host signaling in response to the microbe [examples include references (186–192)] and (ii) attempts to co-culture host cells and *B. burgdorferi* to measure bacterial responses to host cells [examples include references (193–195)]. While the former has had great success in identifying innate immune pathways that contribute to *B. burgdorferi*-induced inflammation, the latter is limited by the different nutrient requirements for *B. burgdorferi* and common cell lines—usually resulting in altered physiology of either the host or microbe.

Beyond transcriptomic approaches, considerable progress has been made in developing tools to understand *B. burgdorferi* behavior during an animal model of infection. This includes intravital imaging of fluorescently labeled bacteria during murine infection (196–200) and luciferase reporter based *in vivo* imaging (130, 201, 202). Additionally, while quantitative PCR (qPCR) or RT-qPCR have long served as the gold standard for quantifying *B. burgdorferi* burden (by DNA or RNA, accordingly), we note that approaches using either luciferase-based (201) or digital-droplet PCR-based (203) approaches are becoming increasingly common.

## SELECTED INSIGHTS FROM THE USE OF *B. BURGDORFERI* MODELS OF INFECTION

The advances chronicled above have yielded a powerful set of tools for dissecting host–pathogen interactions in Lyme disease. In the following sections, we will discuss how the different models of *B. burgdorferi* infection have been used to reveal the fundamental biology of this organism. Space limitations prevent us from detailing all the studies resulting from the use of these models, so we will focus on just a few key thematic elements of *B. burgdorferi* pathogenesis.

### Shifting transcriptomic profiles allows environmental adaptation and survival in different hosts

Considerable attention has been paid to how *B. burgdorferi* gene expression is regulated across different hosts [reviewed in reference (204, 205)]. An early discovery was that *B. burgdorferi* expresses the outer surface protein *ospA* in unfed ticks, but the population shifts to begin expressing the outer surface protein *ospC* following the blood meal (173). This mimics the requirements for each protein: *ospA* is required for the colonization of ticks (206, 207), while *ospC* is required for mammalian infection (208). This can be recapitulated *in vitro* as OspC protein expression is higher in BSK-media when temperature is shifted to 37°C and downregulated when shifted to lower temperatures (173). Further replicating *in vivo* studies (209), single-cell studies using flow cytometry have demonstrated that a lower proportion of spirochetes express OspA at 37°C, while some spirochetes remain OspA^+^OspC^-^ or OspA^+^OspC^+^ at high temperatures—though examining of RNA abundance in these OspA^+^ spirochetes suggest that they are beginning to downregulate *ospA* (210). These expression patterns of *ospA* and *ospC* established a paradigm with which to understand host-specific gene expression *in B. burgdorferi*. As the tools for manipulating *B. burgdorferi* improved and the ability to identify gene regulation in native hosts expanded, it became clear that OspA and OspC were just two components of larger networks of gene regulation responding to environmental conditions.

A primary gatekeeper of vertebrate-associated gene expression, including *ospC*, in *B. burgdorferi* is the alternative sigma factor RpoS, which itself is transcriptionally activated by a second alternative sigma factor RpoN (178, 211). *rpoS* is induced during tick feeding (183) and is required for successful migration from the tick midgut to the salivary glands for infection of the vertebrate host (185, 212). This process is, perhaps unsurprisingly, activated by numerous factors, most notably Rrp2 (BB0763) (213) and BosR (BB0647) (214–216). Completing the loop, RpoS is also able to suppress tick-associated gene expression, including *ospA* (217), by binding to the promoter region (218). Interestingly, while recent work has shown that BosR positively regulates RpoS by stabilizing RNA transcripts (219), the protein may also serve to directly repress *ospA* genes by binding near the *ospAB* promoter (220). Notably, while critical, RpoN-RpoS make up only one of three regulatory systems that facilitate appropriate gene signaling throughout the enzootic cycle. The cyclic dimeric GMP (c-di-GMP) producing Hk1/Rrp1 two-component system (221–223) and the *Rel_Bbu_*/*dksA*-mediated stringent response (224–227) also facilitate survival in rodent and/or tick hosts. Interestingly, these pathways appear to feedback into one another, as Rrp1/c-di-GMP (228, 229) and Rel_Bbu_ (225, 227) regulate RpoS.

Numerous models have been proposed for how these proteins work in concert to sense which host the spirochete is preparing to inhabit, and here, we will focus on two non-mutually exclusive hypotheses. First, DNA supercoiling at lower temperatures (such as in ticks) is thought to promote *ospA* and impair *ospC* expression (230). However, shifting unfed, *B. burgdorferi*-colonized nymphs to high temperatures (37°C) is insufficient to induce *ospC* expression in the midgut (173), demonstrating that this cannot be the only mechanism at play. Second, *B. burgdorferi* are able to sense their replication rate—which is informed by nutrients and temperature, two major signals that differentiate a starved and feeding tick, to regulate OspC expression (231)—though growth rate does not appear to regulate *ospA* expression. While we note that these hypotheses are well supported, additional levels of *ospA* and *ospC* regulation are possible, if not probable.

### *B. burgdorferi* parasitizes host processes during colonization

In addition to encoding a limited number of metabolic processes, the *B. burgdorferi* genome has only a small toolkit for interfacing with a host and indeed lacks traditional virulence factors. Despite this, the spirochete takes advantage of invertebrate and vertebrate host processes to facilitate its enzootic cycle.

In order to escape the tick and enter a new host, *B. burgdorferi* must depart the tick midgut, where it resides after colonizing a larval tick, and make its way to the salivary glands (232–234). Live-cell fluorescent microscopy studies during the nymphal blood meal show a “biphasic mode of dissemination” in which non-motile, proliferating *B. burgdorferi* penetrate the epithelium before transitioning to a motile state capable of penetrating the basement layer into the hemocoel (the body compartment in ticks containing hemolymph) and swimming to the salivary glands (235). From there, the bacteria enter the vertebrate host as the tick injects its saliva during feeding. After arriving in the vertebrate, host *B. burgdorferi* must contend with a hostile environment as the wound from the tick bite site recruits neutrophils in rodents (236, 237) and humans (238). In order to survive this initial introduction to the vertebrate innate immune system, *B. burgdorferi* takes advantage of proteins secreted by *I. scapularis* to modulate the host immune response (239). *I. scapularis* secrete these proteins to enable a stealthy and prolonged blood meal, but these proteins are also utilized by *B. burgdorferi*. Indeed, early studies demonstrated that simply co-injecting tick saliva and *B. burgdorferi* into mice gave the bacteria a substantial colonization advantage (240), though curiously this is highly species specific—*I*. *scapularis* saliva protected *B. burgdorferi* but not *Borrelia lusitaniae*, while *Ixodes ricinus* saliva protected *B. lusitaniae* without protecting *B. burgdorferi*. A recent *ex vivo* study demonstrated that tick saliva actively suppressed human macrophage and neutrophil migration toward invading *B. burgdorferi*, which correlated with increased *B. burgdorferi* density (238). More mechanistic studies have identified roles for numerous saliva proteins in promoting *B. burgdorferi* transmission, including Salp15-mediated protection from serum (241, 242) and CD4 T cell activation (243), IxsS17- and TSLPI-mediated protection from complement (244, 245), and IsC1ql3-mediated suppression of the interferon-γ response (246). Together, these co-opted components of tick saliva enable the initial *B. burgdorferi* landing party to successfully exit the tick and spread into the mammalian host tissue.

A major danger to *B. burgdorferi* during both early- and late-vertebrate infection is the antibody response paired with the complement cascade. Interestingly, this threat begins even before transitioning into the vertebrates and extends after exiting back into the tick, as antibody and complement within the tick blood meal can bind and kill *B. burgdorferi*. The ability of antibody to kill *B. burgdorferi* in ticks appears to be both complement dependent and independent—complement kills antibody-bound *B. burgdorferi* in larval ticks but is not thought to be required for the bactericidal activity in nymphal ticks (247). *B. burgdorferi* complement-independent antibody killing is thought to occur by osmolytic stress (248).

While there are many ways *B. burgdorferi* resists complement [recently reviewed in reference (249)], one notable way it escapes destruction is by co-opting the host complement inhibitor, Factor H, during pathogenesis (250–254). Interesting recent work has examined a hypothesis that different reservoir tropisms across different *Borrelia* species could be credited to differences in the ability of each *Borrelia* species to resist complement in preferred hosts—in part through factor H binding [reviewed in reference (255)]. A recent study by Marcinkiewicz et al. provided mechanistic data to support this hypothesis, demonstrating that natural variation in CspZ (BBH06) directly impacts the ability of *B. burgdorferi* to bind mouse or quail complement inhibiting protein factor H and survive in the blood of either species (256). Similarly, new work on eastern fence lizards (*Sceloporus undulatus*), a relatively rare host for the spirochete, found that while the reptilian complement was extremely potent at killing *B. burgdorferi*, a small number of strains were able to survive following exposure (257). Using this natural diversity as a launching point, Nowak et al. were able to identify *ospE* variation as contributing to differential survival in the lizard serum via binding of *S. undulatus* factor H. Together, these studies demonstrate that complement likely shapes *B. burgdorferi* evolution and transmission in nature. Furthermore, they raise questions about the evolutionary constraints and directions that *B. burgdorferi* is likely to undergo in the future: specifically, whether a given *B. burgdorferi* lineage is more fit when adapted to a specific host (mammalian, avian, or reptilian) or is more advantaged remaining as a generalist. The answer to this question likely depends on a variety of biotic and abiotic factors, which may differ across geographical space. This makes *B. burgdorferi* an excellent model to understand host-microbe co-evolution and adaptation.

### *B. burgdorferi* hides from host immunity to establish long-term infection

The lack of known virulence factors means that *B. burgdorferi* has a limited arsenal for attacking host immune responses directly. But, as an organism that is able to persist in its tick and vertebrate hosts for long periods of time, it has evolved an array of tactics for evading or outsmarting host immunity. First, these speedy spirochetes [for motility in the skin, see reference (196), Movie S1; for motility in the dura mater, see reference (91), Movie S1] are dramatically faster than neutrophils (which are faster than most immune cells) (258), making them difficult to actually catch and phagocytose. To the bacteria’s benefit, this likely synergizes with the immune response shifting from a neutrophil-dominant response (~6 hours post-infection) to a slower-moving macrophage-dominant response (~16 hours post-infection) in mice (259). These findings are supported by human reports in which the erythema migrans rash appears enriched for macrophages but depleted of neutrophils (260). While *B. burgdorferi* can escape early immune responses upon entry, this does not mean every spirochete does. Disruption of innate immunity [e.g., TLR2 knockout (186) and MyD88 knockout (261)] leads to orders of magnitude higher *B. burgdorferi* burdens in mice during early infection, demonstrating that many, if not most, spirochetes are successfully cleared by the innate immune response.

Upon establishing infection in a new vertebrate host, the bacteria downshift into stealth mode and begin to downregulate many of their outer surface proteins, going from expressing >100 lipoproteins during the first 10 days of infection to expressing fewer than 40 lipoproteins by 33 days post infection (181). A plausible hypothesis is that this downregulation provides the immune system with fewer targets for antibodies. This is supported by data that have shown that while OspC is absolutely required for early murine infection, the bacteria need to downregulate the expression of the protein during late infection, and constitutive *ospC* expression results in clearance by the adaptive immune system (180, 262).

Another strategy used by *B. burgdorferi* is not just to reduce the number of potential antibody targets but to continually change them (263–265). *B. burgdorferi vlsE* (*bbf0041*) encodes a major lipoprotein that undergoes continuous recombination during mammalian infection (266) but not during *in vitro* cultivation (267). The disruption of the *vls* locus in ways that prevent recombination results in only transient infection in immunocompetent hosts with clearance of the organism once the adaptive immune system responds (268–270). *vlsE* provided on a complementing plasmid in *trans* could not rescue clearance of the pathogen—which overall supports the idea that the actual recombination of this locus is key to antigenic switching and *B. burgdorferi* retention in immunocompetent hosts (268). Recent developments in sequencing have allowed an enhanced understanding of *vlsE* switching, as well as uncovered a role for error-prone DNA replication at the *vls* locus in contributing to antigenic variability (271, 272). The exact role of the *vls* locus and VlsE in immunoevasion is not fully understood (268).

A final strategy used by *B. burgdorferi* to evade host immune systems involves co-opting pathways the host has developed to prevent continual immune activation. In some ways, this draws parallels to host responses to commensal organisms. After initial contact with *B. burgdorferi*, professional immune cells, including macrophages and T cells, are greatly dampened in their responses to *B. burgdorferi* with greater activation of anti-inflammatory rather than proinflammatory pathways (191, 203, 273–276). In this way, *B. burgdorferi* is able to hide from the immune system by being treated as a tissue-invasive “commensal” organism. Additionally, while there is a robust antibody response against *B. burgdorferi*, this response is short-lived following antibiotic treatment in *M. musculus* (277) and overall characterized by a failure to maintain long-lived germinal centers (278, 279). As discussed with the innate immune system, while these evasion methods prevent eradication of the spirochete, studies from severe combined immunodeficiency mice (280) or B cell depleted mice (281) demonstrate that the adaptive immune system is still able to suppress *B. burgdorferi* burden during infection.

## FUTURE DIRECTIONS

### Harnessing *B. burgdorferi* natural diversity

Like all microbial pathogenesis fields, the *B. burgdorferi* community has benefited from selecting a small number of “wild-type” strains—often B31 (the genetic “type” strain), N40, 297, JD1, or Sh-2–82. This has allowed researchers across institutions to more directly compare results, overall making the literature more coherent and facilitating the establishment of a fundamental set of facts for the field (including most of what is listed above). However, there is much to be learned from studying diverse strains of the organism. Cross-species studies have long since revealed that different strains of *Borrelia* are linked to different disease manifestations in humans [e.g., *B. garinii* and neurological disease and *Borrelia afzelli* and late skin infections (282)], and now multi-strain studies have already provided interesting insights into host-specific adaptations of *B. burgdorferi* to specific reservoirs (106, 257) as well as insight into inter-strain competition (283) and subsequent infection dynamics (104, 283, 284). Notably, many of these studies used traditional genotyping of the *ospC* locus (285) to select “representative” strains from different clades of *B. burgdorferi*. However, as larger banks of strains have become available (particularly larger banks of *sequenced* strains), recent work has begun leveraging a wider view of *B. burgdorferi* genetic diversity. One such study tested 11 strains for their ability to colonize and disseminate in C3H/HeJ mice, as well as be aquired by *I. scapularis* (286). A second study sequenced 299 *B. burgdorferi* isolates in an attempt to correlate clinical outcomes with *B. burgdorferi* genotypes (128). These types of diversity studies will likely be critical to enhancing our understanding of both how *B. burgdorferi* spreads in nature and how it causes illness in human patients. We expect that the use of a diverse population of strains for study will become standard in the coming years.

### Determine the frequency and impacts of stochastic heterogeneity in *B. burgdorferi*

Interestingly, not all diversity in *B. burgdorferi* is genetically based. Heterogeneity in gene expression is commonly seen in *B. burgdorferi* that is (presumably) genetically identical. How and why this diversity is generated is not well understood. For example, upregulation of OspC and downregulation of OspA in response to temperature *in vitro* do not occur equally across clonal cells, as demonstrated by flow cytometric measurements of Osp expression (210). We note that while DNA methylation has historically been a popular hypothesis for non-genetically encoded natural diversity in other species (287), the search for widespread impacts of DNA methylation on the *B. burgdorferi* transcriptome has yielded variable results (288, 289), and we are unaware of any study that has specifically attempted to associate methylation and heterogeneity in gene expression. Regardless of the mechanism, it is tempting to hypothesize that generation of non-genetic diversity may be beneficial to an organism that traverses many different hosts and requires different strategies to survive in hosts as diverse as birds, lizards, and rodents.

### Probing *B. burgdorferi*-host interactions in infected tissues

As discussed above, considerable work has been done examining host-pathogen interactions *in vitro* or *in vivo*, but the vast majority of these techniques have examined interactions in bulk, e.g., what host or bacterial genes, pathways, or cells influence *B. burgdorferi* survival or burden. While these studies have been extremely important in understanding *B. burgdorferi* pathogenesis, they ignore the relatively complex interactions that *B. burgdorferi* has with cells as a motile extracellular pathogen. Little is known about how host cells (immune cells, fibroblasts, keratinocytes, cardiomyocytes, and synoviocytes) that directly interact with *B. burgdorferi* respond to the pathogen, or how neighboring “bystander cells” react to the changes this causes in the tissue microenvironment. While these studies are technically challenging, technological advances are paving the path for these experiments to become possible. First, as discussed above, there have been numerous successful attempts to visualize *B. burgdorferi* in mice using live imaging (91, 196–200). These studies could be linked with fluorescent reporters of host gene expression to dissect some of these questions using live imaging. Additionally, the dissection of *B. burgdorferi* infected tissues and the use of spatial transcriptomics (290) could allow an enhanced understanding of heterogeneity in cellular responses to *B. burgdorferi*.

### Generating a broader understanding of bacterial cell biology

As covered in another review in this series (291), there is a need for “‘non-model’ model bacterial systems.” While the ability of *E. coli* to model bacterial cell and molecular biology has been drawn into question in numerous fields [even some genetic circuits between *E. coli* and the closely related *Salmonella* genus show a high degree of divergence (292, 293)], the ability to model *B. burgdorferi* using *E. coli* can perhaps best be summarized by the immortal words of Dr. Ben Adler “Spirochetes do it differently!” There is considerable interest in understanding how metabolism, motility, protein secretion, chromosome segregation, replication, cellular growth, and numerous other base processes occur in *B. burgdorferi* that will only be answered through continued basic science studies utilizing the microbe itself. We note that while there is hope that understanding how these processes occur in *B. burgdorferi* will illuminate how they occur in other spirochetes (particularly *Leptospira* and *Treponema* species), the unique aspects of *B. burgdorferi* genome organization (7–9, 294) and its biphasic host-associated lifestyle may drive specific adaptations to some of these universal biological problems.

### Harnessing the awesome power of human genetics

Finally, we note that while natural genetic diversity in mouse strains has been leveraged to understand genes that contribute to different disease outcomes following *B. burgdorferi* infection (93, 94), few studies have successfully identified human genetic variants that contribute to Lyme disease outcomes. Appropriate use of human genetics can not only explain natural diversity in disease outcomes but also reveal molecular mechanisms of pathogenesis and identify targets for potential therapeutic interventions (295). One targeted genetic study identified the single nucleotide polymorphism rs5743618, which results in an amino acid change in the Toll-like receptor 1 (*TLR1*), that associated with susceptibility for antibiotic refractory Lyme arthritis (192). Recently, the first human genome-wide association study (GWAS) was published, which identified three loci that associate with Lyme disease: rs9276610 in the HLA locus, the *TLR1/6/10* expression quantitative trait locus rs17616434, and a missense variant rs2232950 in Secretoglobin family 1D member 2 (*SCGB1D2*) (56). Follow-up studies confirmed that SCGB1D2 has antimicrobial properties that are reduced by the rs2232950 risk allele, which results in a leucine at amino acid position 53 of SCGB1D2, providing a potential mechanism for the genetic association data. A second GWAS published shortly later replicated the rs2232950 hit and identified rs1061632—an expression quantitative trait locus for *KCTD20* and *ETV7*, as associated with Lyme borreliosis (57). While these studies shine new light on human susceptibility to Lyme disease, there are likely many more common genetic variants that contribute to susceptibility and severity during infection, and thus, additional study of natural human diversity during *B. burgdorferi* infection is warranted.
